# Miniaturized Frustum-Cone Triboelectric Hydrophone Based on a Thin Film Perforated Tube Structure

**DOI:** 10.3390/nano15231765

**Published:** 2025-11-25

**Authors:** Yufen Wu, Jing Liu, Yanling Li, Xin Na, Wei Qiu, Qiang Tan

**Affiliations:** 1 College of Physics and Electronic Engineering, Chongqing Normal University, Chongqing 401331, China; 2 College of Optoelectronic Engineering, Chongqing University, Chongqing 401120, China; liujing@cqipu.edu.cn (J.L.); 202508159580@stu.cqu.edu.cn (Y.L.); 3 Chongqing Municipal Water Supply Co., Ltd., Chongqing 400060, China; cqnaxin@163.com (X.N.); cqzlsqw@163.com (W.Q.); tanqiang727@163.com (Q.T.)

**Keywords:** low-frequency hydroacoustic detection, coupled membrane-cavity structure, triboelectric nanogenerator, triboelectric hydrophone

## Abstract

Underwater acoustics is the optimal method for long-distance information transmission in aquatic environments. Hydrophones, as the core component of sonar systems, have found widespread application across multiple fields. However, existing types of hydrophones exhibit limited detection capabilities under low-signal conditions. To enhance low-frequency long-range detection performance, the development of new hydrophones featuring low power consumption, low frequency, high sensitivity, and miniaturization has become a research priority, with breakthroughs sought in the principle of electroacoustic conversion. Therefore, this study designed a frustum-cone triboelectric hydrophone (FCTH) based on friction layer materials, utilizing an indium-tin oxide (ITO) flexible conductive film on a polyethylene terephthalate (PET) substrate and a Polytetrafluoroethylene (PTFE) film. The sensor consists of a waterproof, sound-transparent polyurethane flow guide, silicone oil, and a frustum-cone triboelectric sensing unit based on a coupled membrane–cavity structure. The frustum-cone triboelectric sensing unit, based on a thin-film-perforated-tube resonance structure, enables omnidirectional detection of low-frequency hydroacoustic signals. The miniaturized design significantly reduces the volume of the FCTH. The acoustic–electric conversion relationship of the FCTH was derived using acoustic theory, thin-film vibration theory, and Maxwell’s displacement current theory. Furthermore, the low-frequency response characteristics of the frustum-cone triboelectric sensing unit were analyzed. The FCTH achieves a wide-frequency response ranging from 50 Hz to 12,000 Hz, with omnidirectional sensitivity and a maximum sensitivity of −174.6 dB. The FCTH achieves a wide-frequency response capability of 50 Hz to 12,000 Hz, with omnidirectional sensitivity and a maximum sensitivity of −174.6 dB. Additionally, through acoustic signal acquisition experiments in air, indoor, and outdoor water environments, the FCTH has been validated to possess excellent underwater acoustic detection performance and application potential across multiple scenarios.

## 1. Introduction

The ocean is rich in resources and serves as the material foundation for human survival and sustainable social development [1,2,3,4]. Since the 21st century, countries around the world have invested significant human and material resources to enhance their capabilities in exploring and developing marine resources, promoting marine economic development, and strengthening marine scientific and technological capabilities. As demands for marine environmental safety continue to rise, efficiently obtaining underwater information has become a key factor in advancing marine detection technology. Due to the severe attenuation of electromagnetic waves in water, sound waves are currently the optimal medium for transmitting energy and information underwater, offering advantages such as long transmission distances, high stability, and immunity to interference from light, electric fields, and magnetic fields [5,6,7]. Sonar systems are essential devices for detecting underwater acoustic information, widely applied in underwater communication, target identification and tracking, seabed resource exploration, marine animal research, and military weaponry [8,9,10,11]. Hydrophones are the core components of sonar detection equipment, converting acoustic signals into electrical signals. Therefore, the acoustic detection capability of sonar systems is closely tied to the performance of hydrophones.

With the growing demand for modern marine technology, underwater acoustic detection technology has entered a new era. Research has shown that the sound waves generated by marine activities such as earthquakes, tsunamis, and volcanic eruptions typically have frequencies below 20 Hz. The inherent noise from submarines, fishing vessels, and other ships primarily concentrates in the low-frequency range of 50 Hz to 500 Hz. Therefore, high-sensitivity low-frequency hydrophones are increasingly valuable in fields such as underwater earthquake monitoring, tsunami monitoring, nuclear explosion monitoring, and submarine detection [12,13,14,15,16]. Additionally, the emergence of nuclear submarines and underwater vibration reduction and noise suppression technologies have posed significant anti-submarine challenges for sonar systems. Nuclear submarines can launch long-range missiles and have excellent stealth capabilities, making them difficult to detect. With the support of underwater vibration reduction and noise suppression technologies, such as sound-absorbing tiles, the noise power of nuclear submarines has decreased year by year, and their noise spectra have increasingly concentrated in the low-frequency range [17,18,19,20]. Compared to high-frequency underwater acoustic signals, low-frequency signals experience less propagation attenuation and can effectively transmit over distances of thousands of kilometers. Therefore, developing low-power, low-frequency, high-sensitivity, and miniaturized hydrophones has become a research priority [21,22]. In current low-frequency underwater acoustic detection applications, piezoelectric hydrophones [23,24,25] and fiber-optic hydrophones [26,27,28] dominate the market due to their relatively mature manufacturing technology and high sensitivity. However, existing hydrophones still lack sufficient sensitivity when detecting low-frequency underwater acoustic signals ranging from tens to hundreds of hertz, failing to meet the rapidly evolving needs of modern underwater acoustic sensing technology. Specifically, due to the effects of extended signal lines and parasitic impedance, the high impedance of piezoelectric materials causes piezoelectric hydrophones to lose some data and experience reduced sensitivity during low-frequency response, particularly in the frequency range of tens to hundreds of hertz, where piezoelectric hydrophones exhibit lower sensitivity compared to other operating frequency bands [29]. Fiber-optic hydrophones have complex manufacturing processes and typically include preamplifiers, making their sensitivity difficult to directly compare [30]. Therefore, the development of a new type of low-frequency hydrophone holds significant practical importance.

Triboelectricity, a phenomenon commonly observed in daily life, is often overlooked as a negative effect and not effectively utilized. However, in recent years, triboelectric nanogenerators based on the principle of triboelectricity coupled with electrostatic induction have garnered significant attention due to their low cost, simple structure, high energy conversion efficiency, and flexible application scenarios [31,32,33]. By reasonably designing the sensor structure, triboelectric nanogenerators can collect various irregular mechanical energies generated during friction and convert them into electrical energy. For example, they can convert green energy sources such as wind energy, rain energy, and wave energy into electrical energy for storage and power generation [34,35,36,37,38,39,40,41]. Additionally, by collecting kinetic energy from friction in daily human activities, triboelectric nanogenerators can also function as mechanical sensors for applications in human health monitoring, human–machine interaction, and self-powered biomedical devices [42,43,44,45,46,47,48,49,50,51]. As a form of mechanical vibration, sound waves also transmit mechanical energy. Therefore, sensors based on triboelectric nanogenerator technology can also collect sound signals and convert them into electrical signals. Currently, there have been studies on triboelectric sensors for voice signal collection in related reports [52,53,54], but there are few reports on hydrophones based on the triboelectric effect. Therefore, given the high sensitivity of triboelectric sensors in perceiving low-frequency, irregular mechanical energy, this study investigated triboelectric low-frequency sound pressure hydrophones, which could provide new research directions for underwater acoustic detection technology.

To achieve the design objectives of miniaturization, omnidirectionality, low frequency, and high sensitivity, this study proposes a frustum-cone triboelectric hydrophone (FCTH). The device has a frustum cone structure. It is designed with a thin film and a perforated tube to form a resonant structure. The frustum-cone triboelectric hydrophone consists of a cylindrical polyurethane flow guide, silicone oil, and a frustum-cone triboelectric sensing unit. The friction layer materials selected are a Polytetrafluoroethylene (PTFE) thin film and an indium-tin oxide (ITO) conductive thin film based on a polyethylene terephthalate (PET) substrate. Combining acoustic principles, the triboelectric effect, and thin-film vibration theory, the mechanical and electrical characteristics of the FCTH were analyzed. Simulation analysis was conducted on the FCTH, and its sensing characteristic parameters, such as frequency response range, sensitivity, and directional stability, were tested. Additionally, acoustic signal acquisition tests were designed in different environments to validate the underwater acoustic detection capabilities of the FCTH.

## 2. Materials and Methods

### 2.1. Structure of the FCTH

**Figure 1:**
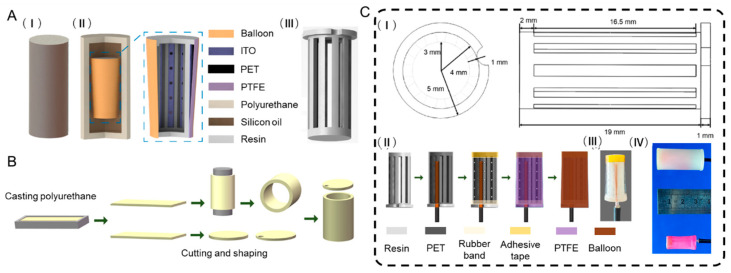
(**A**) Design of the FCTH: (**I**) The round table triboelectric hydrophone; (**II**) Structure diagram of the FCTH; (**III**) Cylindrical hollow frame. (**B**) Preparation process diagram of the cylindrical dome. (**C**) The structural dimensions of the frustum-cone triboelectric sensing unit: (**I**) Dimensions of a cylindrical frame; (**II**) Preparation process diagram of the frustum-cone triboelectric sensing unit; (**III**) Physical picture of the frustum-cone triboelectric sensing unit. (**IV**) Physical view of the frustum-cone triboelectric sensing unit and the FCTH.

This study designed a frustum-cone triboelectric hydrophone based on a thin-film-perforated-tube resonant structure. As shown in Figure 1A(I,II), the FCTH features an overall cylindrical structure comprised silicone-oil acoustic coupling layer, a cylindrical polyurethane flow guide, and a frustum-cone triboelectric hydroacoustic transducer unit based on a thin-film-perforated-tube coupled resonant structure. The friction layer materials for the frustum-cone triboelectric hydroacoustic transducer unit are PTFE ultra-thin film and PET film, operating in a single-electrode contact-separation mode. The PET film side is coated with conductive ITO, and electrical signals are output by connecting metal wires to the ITO conductive coating. To meet the development requirements for omnidirectional and miniaturized hydrophones, a hollow cylindrical frame was designed with differing diameters at its upper and lower ends, as shown in Figure 1A(III). The PET film is wrapped around the sides of the hollow cylindrical frame, while the PTFE film is bonded to its upper and lower ends. By bending the contact surfaces of the friction materials, the volume of the triboelectric hydrophone is reduced while achieving the design goal of omnidirectionality. Simultaneously, an inclined air gap layer forms between the PTFE and PET films. This gap must be sufficiently narrow to induce contact separation while providing adequate space for the membrane cavity coupling resonator to function properly. A circumferential array of perforations on the PET film connects the gas within the hollow cylindrical frame to the air gap of the friction layer, forming a film-perforated tube resonator structure. Furthermore, to prevent acoustic coupling agent from permeating into the air gap layer, the PTFE film is encapsulated within a natural latex balloon.

### 2.2. Fabrication of the FCTH

#### 2.2.1. Preparation of the Cylindrical Dome

Based on the principle of acoustic impedance matching, a cylindrical guide hood was fabricated using castable polyurethane resin. The guide hood has an outer diameter of 17 mm, a thickness of 2 mm, and a total height of 35 mm. A 3 mm diameter hole was drilled into one side of the polyurethane disc. Figure 1B illustrates the fabrication process of the cylindrical flow guide. First, a polyurethane release agent is sprayed onto the inner surface of the silicone mold. Next, the liquid casting polyurethane components, P6-A and P6-B, are thoroughly mixed at a 1:1 mass ratio and then poured into the mold. The cast polyurethane mixture requires approximately 1 h to fully cure. After 20 min of curing in the silicone mold, the partially cured polyurethane block is demolded for trimming and shaping. Finally, a polyurethane disc is bonded to the cylindrical polyurethane shell using epoxy adhesive to form a single-ended open cylindrical housing. A circular hole is drilled at the center of another polyurethane disc to reserve space for the metal wire.

#### 2.2.2. Fabrication of the Frustum-Cone Triboelectric Sensing Unit

The materials for fabricating the FCTH primarily include: a hollow cylindrical frame, PET film coated with an ITO conductive layer, PTFE film, transparent rubber bands, metal wires, and natural latex balloons. Considering that the FCTH requires perforations in the triboelectric layer, opaque metallic materials are unsuitable for use as either the triboelectric layer or the electrode layer. Therefore, PET film and PTFE film were selected as triboelectric layer materials due to their advantages of being easily bendable, easily cut, coated with an ITO conductive layer, and color-transparent.

To further increase the effective contact area of the friction layer and enhance the output electrical signal, nanowire arrays were fabricated on the surface of PTFE film. The fabrication process is as follows: First, the PTFE membrane was cleaned with menthol, isopropanol, and deionized water. The dried PTFE membrane was then placed into the reaction chamber of the reactive ion etcher. After closing the chamber door, experimental parameters were set: oxygen gas flow at 30 sccm, RF power at 100 W, and a process duration of 5 min. Vacuum pumping commenced, and etching began when the ionization unit reached 6–2 Pa, yielding the etched PTFE membrane. The SEM image is shown in Figure S1.

Using 3D printing technology, a resin hollow cylindrical frame was fabricated according to the dimensions in Figure 1C(I). Its upper outer diameter is 10 mm, lower outer diameter is 8 mm, and total height is 20 mm. A groove is reserved at the top of the hollow cylinder to accommodate wire placement. Commercially available ITO-coated PET film, with a thickness of 0.05 mm and a surface resistance of 150 Ω, was used. The fabrication process of the FCTH is illustrated in Figure 1C(II). First, metal wires were threaded through circular holes in polyurethane discs and adhered to the ITO coating. Subsequently, the PET film with attached metal wires was affixed to the entire lateral surface of the hollow cylindrical frame, positioning the wires precisely within the semicircular groove at the top of the hollow cylinder. To address potential adhesion issues, the PET film ends were reinforced: the top was secured with 0.05 mm thick adhesive tape, while the bottom was fastened using an 8 mm diameter, 1 mm wide transparent rubber band. Subsequently, a circumferential array of air holes was prepared on the PET film, arranged in 10 columns of 5 holes each, spaced 2 mm apart. Finally, the 0.05 mm PTFE film was stretched taut and adhered to the top and bottom ends of the cylindrical frame. After placing a balloon over the assembly, the top and bottom ends of the frustum were sealed with epoxy resin adhesive, completing the frustum-cone triboelectric hydroacoustic transducer unit. Figure 1C(III) shows the transducer unit prior to the placement of the latex balloon.

#### 2.2.3. Assembly of the FCTH

The FCTH consists of a cylindrical polyurethane flow guide, silicone oil, and a frustum-cone triboelectric hydroacoustic transducer unit. The frustum-cone triboelectric hydroacoustic transducer unit is secured at the center of the fixed cylindrical polyurethane flow guide and filled with silicone oil. Specifically, epoxy adhesive is used to secure a polyurethane disc previously fitted over a metal wire to the wire. The disc’s radial direction is perpendicular to the wire’s extension, positioning the FCTH precisely at the center of the polyurethane flow guide. Subsequently, the FCTH is placed inside the polyurethane flow guide. Silicone oil is then injected into the polyurethane flow guide shell using a syringe, gradually filling the remaining space within the guide. After ensuring no air bubbles remain in the silicone oil, the polyurethane disc is aligned with the polyurethane cylinder and sealed with epoxy resin adhesive. Finally, a layer of Kraft 705 waterproof adhesive is applied over the fully cured epoxy resin coating to enhance water resistance. Figure 1C(IV) shows the fabricated the FCTH and the assembled hydrophone.

## 3. Results and Discussion

### 3.1. Working Principle of the FCTH

The FCTH consists of two components: the FCTH unit and the waterproof acoustic transmission layer. Based on the principle of hydroacoustic impedance matching, hydroacoustic signals pass through the waterproof acoustic transmission layer to reach the FCTH conversion unit. The resulting sound pressure difference causes the PTFE film and PET film to contact and separate, generating an electrical signal that varies with the acoustic wave. The FCTH employs a single-electrode contact-separation mode, exhibiting the following mechanical and electrical characteristics:

#### 3.1.1. Mechanical Characteristics of the FCTH

**Figure 2:**
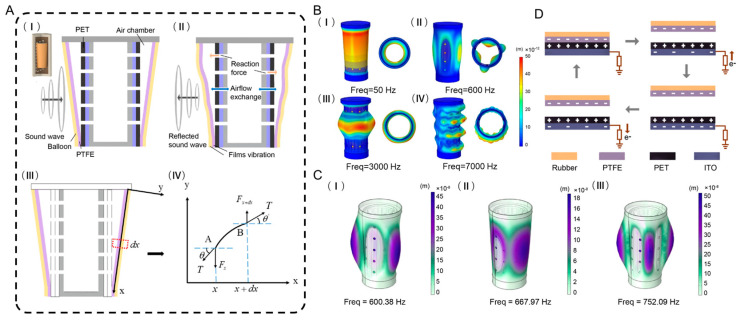
(**A**) Two-dimensional force analysis diagram of the frustum-cone tribological electro-hydroacoustic sensing unit: (**I**) before the arrival of sound wave; (**II**) after the arrival of sound wave; (**III**) and (**IV**) Force analysis diagram of the string element. (**B**) COMSOL simulation results of the frustum-cone triboelectric hydrophone; (**I**) f = 50 Hz; (**II**) f = 600 Hz; (**III**) f = 3000 Hz; (**IV**) f = 7000 Hz. (**C**) Characteristic frequency simulation of the frustum-cone triboelectric hydrophone: (**I**) f = 600.38 Hz; (**II**) f = 667.97 Hz; (**III**) f = 752.09 Hz. (**D**) Charge distribution diagram of the frustum-cone triboelectric hydrophone.

The FCTH employs a conical triboelectric acoustic transducer unit based on a thin-film-perforated-tube resonant structure. The tensioned elastic PTFE film and perforated tube form a resonant system. Before the underwater sound wave arrival, the cross-section of the FCTH unit is as shown in Figure 2A(I,II). Upon reaching the PTFE membrane surface, the acoustic pressure difference between the silicone oil and the air cavity causes a rapid response. The PTFE membrane and the balloon act as elastic materials. They quickly deform under the applied pressure. Simultaneously, air flow and reaction forces occur within the cavity [55], ultimately causing the friction layer materials to contact each other and output an electrical signal, as shown in Figure 2A(II).

Due to the frustum shape of the PTFE membrane and its non-uniform tension, it is impossible to derive a theoretical vibration formula for the entire membrane. Therefore, when calculating the deformation displacement of the frustum-cone PTFE membrane, the non-uniformly tensioned membrane must be simplified into a non-uniformly tensioned chord for vibration analysis. The vibration displacement of the chord element is used to represent the membrane’s vibration state.

Assume the non-uniformly tensioned chord has length l, with its initial position coinciding with the x-axis and vertical displacement η=0. Select a chord segment with projection dx on this non-uniformly tensioned chord, whose ends are labeled x and x+dx, as shown in Figure 2A(III,IV). Since dx is very small, the tension along this chord segment can be considered uniform. Let the tension along this segment be denoted as T. When the chord undergoes forced vibration, the chord segment at x experiences a vertical displacement. Assuming this displacement is η, the resultant vertical tension force on the chord segment is [56]:

(1) $$xdF_{x}=T\frac{\partial^{2}\eta }{\partial x^{2}}dx.$$

In the formula, θ represents the angle between the tangent direction of the chord element at the x-coordinate point and the horizontal direction, which is a function of x.

Under the combined action of the external sound pressure force and the cavity reaction force, the vibration equation of the string element can be calculated based on Newton’s Second Law:

(2) $$T\frac{\partial^{2}\eta }{\partial x^{2}}dx-P_{a}e^{j\omega t}dx+R\frac{\partial \eta }{\partial t}dx=\left(\delta dx\right)\frac{\partial^{2}\eta }{\partial t^{2}}$$

In the equation, δ is the unit length mass of the string element, $P_{a}$ is the sound pressure amplitude in N/m^2^, ω is the angular frequency of the sound pressure, and R is the surface resistance coefficient of the air medium.

Let $c^{2}=T/\delta$, then the vibration equation of the chord element can be simplified to:

(3) $$\frac{\partial^{2}\eta }{\partial x^{2}}=\frac{1}{c^{2}}\frac{\partial^{2}\eta }{\partial t^{2}}-\frac{R}{c^{2}\delta }\frac{\partial \eta }{\partial t}+\frac{P_{a}e^{j\omega t}}{c^{2}\delta }$$

Since the chord segment can be arbitrarily selected, Equation (3) can be used to describe the motion law at any position along a string with non-uniform tension.

Ultimately, the vertical displacement η(x,t) of the chord segment can be determined as:

(4) $$\eta (x,t)=\int_{0}^{t}(C_{1}e^{\lambda x}+C_{2}e^{-\lambda x})[C_{3}e^{\left(\frac{R}{2c^{2}\delta }+\sqrt{\frac{R^{2}}{4c^{4}\delta^{2}}-\lambda^{2}c^{2}}\right)(t-\tau )}+{C_{4}e}^{\left(\frac{R}{2c^{2}\delta }-\sqrt{\frac{R^{2}}{4c^{4}\delta^{2}}-\lambda^{2}c^{2}}\right)(t-\tau )}$$

In the equation, $C_{1}$, $C_{2}$, $C_{3}$ and $C_{4}$ are constant coefficients that can be determined from the boundary conditions and initial conditions.

To obtain the deformation of PTFE membranes under planar acoustic wave stimulation at different frequencies, a COMSOL Multiphysics 6.1 finite element simulation analysis was conducted on the mechanical properties of the FCTH. To simulate the underwater response of the FCTH, a cylindrical water domain with fully absorbed boundaries was added. Materials were configured based on real material properties to effectively avoid acoustic reflection interference and accurately describe its vibration response characteristics. A planar sinusoidal wave propagated from one side, with its propagation direction perpendicular to the central axis of the FCTH. The sound pressure was set to 1 Pa, and the hollow cylindrical frame was fixed. By adjusting the acoustic frequency parameters and initiating a scan, the deformation patterns of the conical PTFE membrane at different frequencies were obtained. To demonstrate the multiple vibration modes of the PTFE membrane under acoustic excitation, Figure 2B(I–IV) show the simulated vibration patterns of the PTFE membrane at acoustic frequencies of 50 Hz, 600 Hz, 3000 Hz, and 7000 Hz, respectively. As the acoustic frequency increases, the vibration patterns become more complex and exhibit reduced symmetry. However, the deformation of the PTFE membrane demonstrates that the FCTH can detect underwater acoustic signals through the membrane.

By selecting the Eigenfrequency Study in COMSOL, the eigenfrequencies of the FCTH can be obtained. Figure 2C(I–III) displays the mode shapes of the PTFE membrane at the first to third eigenfrequencies, which are f = 600.38 Hz, f = 667.97 Hz, and f = 752.09 Hz, respectively. Due to the non-parallel arrangement of the friction layers, the resonant frequency of the FCTH does not necessarily correspond to the maximum output voltage signal at that frequency. Therefore, the characteristic frequency simulation results for the FCTH are provided for reference only; actual measured output values should be considered definitive.

#### 3.1.2. Electrical Characteristics of the FCTH

The FCTH operates in a single-electrode contact-separation mode, converting acoustic signals into electrical signals through effective contact between PTFE and PET films. The open-circuit voltage of the FCTH can be expressed as:

(5) $$V_{OC}(g=\infty )=\frac{Q}{2C_{1}}$$

In the equation, the distance between the reference electrode and the main electrode is g, the total friction charge is Q, and the equivalent capacitance between the ITO electrode and the PTFE film is $C_{1}$.

The charge distribution between the PET film and PTFE film during a complete power generation cycle is shown in Figure 2D. The acoustic pressure difference causes the PET film and PTFE film to come into contact with each other. Since the PTFE film exhibits a stronger attraction to electrons, an equal amount of positive charge and negative charge is generated on the surfaces of the PET film and PTFE film, respectively. When the PET and PTFE films begin to separate, electrostatic induction creates a potential difference between the ITO electrode and the reference electrode. This potential difference drives electrons from the reference electrode (ground) toward the ITO electrode layer until the distance between them reaches its maximum. When the two friction layers approach again, electrons flow back from the ITO electrode layer to ground to balance the new potential difference.

### 3.2. Performance Testing of the FCTH

**Figure 3:**
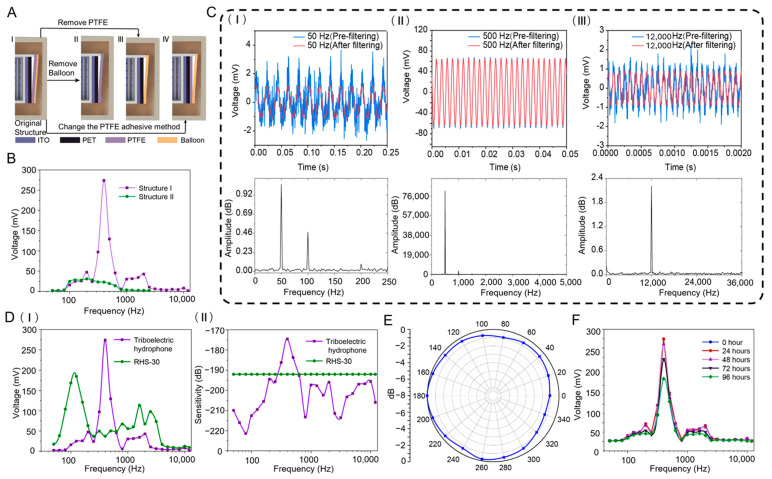
(**A**) Schematic diagram of four frustum-cone triboelectric hydrophones. (**B**) Frequency response curves of frustum-cone triboelectric hydrophones (structure **I** and **II**). (**C**) Output voltage and FFT image of frustum-cone triboelectric hydrophone: (**I**) f = 50 Hz; (**II**) f = 500 Hz; (**III**) f = 12000 Hz. (**D**) Test result curves: (**I**) Frequency response curves of the triboelectric hydrophone and the RHS-30 hydrophone. (**II**) Sensitivity curves of the triboelectric hydrophone and the RHS-30 hydrophone. (**E**) Directivity diagram of frustum-cone triboelectric hydrophone. (**F**) Stability curve of the frustum-cone triboelectric hydrophone.

The FCTH employs a non-parallel friction layer structure, making the air gap height critical to its output performance. To enhance the output performance of the hydrophone, this study designed and compared four FCTH structures, designated as Structure I, Structure II, Structure III, and Structure IV (as shown in Figure 3A).

To ensure interpretable comparison results, a single-factor experimental design was employed. For each structure, only one key parameter was altered while all other structural dimensions, materials, and experimental conditions remained consistent. This approach sequentially verified the impact of different structural factors on output performance.

(1) Structure I: The baseline design features a PTFE film stretched taut and bonded to the upper and lower ends of a hollow cylindrical frame, creating an air gap layer with varying height. A latex balloon is then overlaid to form a double-layer membrane structure.

(2) Structure II: Based on Structure I, the balloon is removed to verify the role of the double-layer membrane structure. The PTFE film joints are sealed with an adhesive layer, while all other parameters remain unchanged.

(3) Structure III: Based on Structure I, only the PTFE membrane is removed while retaining the latex balloon as a new friction layer material to evaluate the impact of friction material type on performance;

(4) Structure IV: Based on Structure I, only the PTFE membrane fixation method is altered—it is no longer tensioned and instead directly covers the PET film surface, thereby disrupting the tensioned membrane–resonant cavity structure to validate the functional role of this design.

Through this single-variable control design, the effects of the latex balloon layer, PTFE membrane material, and membrane tension state on sensing performance can be analyzed independently, avoiding interference from multi-factor coupling.

After testing the frequency response ranges of the four frustum-cone triboelectric hydrophones, results showed that Structures I and II generated voltage signals under sinusoidal acoustic excitation. However, Structures III and IV only produced voltage signals when the sensors themselves were tapped, rendering them ineffective for detecting hydroacoustic signals. Structure III demonstrates that the combination of the latex balloon and the PET film as friction materials is ineffective for detecting low-frequency underwater acoustic signals. Structure IV confirms that detecting underwater acoustic signals via the triboelectric effect requires integrating the forced vibration characteristics of a tensioned film with a resonant cavity structure. Table 1 displays the filtered output voltage values for Structures I and II. For direct comparison, the frequency response curves of Structures I and II are shown in Figure 3B. Structure I exhibits a frequency response range of 50 Hz to 12,000 Hz with a maximum output voltage amplitude of 274 mV, while Structure II covers 50 Hz to 2500 Hz with a maximum output voltage amplitude of 30.9 mV. Comparing both the frequency response range and voltage amplitude, Structure I outperforms Structure II in all aspects. This demonstrates that the double-layer membrane structure enhances both the frequency response range and output voltage amplitude of the frustum-cone triboelectric hydrophone. Consequently, the frustum-cone triboelectric hydrophone based on Structure I was selected for subsequent experiments.

To analyze whether the triboelectric hydrophone can effectively receive signals, the signal is processed using a fourth order Butterworth bandpass filter and a zero phase forward and reverse filter. The filtered signal is then compared with the original signal, and the Fast Fourier Transform (FFT) of the original signal is examined to determine whether the dominant frequency matches the source frequency. Figure 3C(I–III) shows the raw output voltage waveform, filtered voltage waveform, and FFT plot of the raw voltage waveform for the FCTH at sound frequencies of 50 Hz, 500 Hz, and 12,000 Hz, respectively. When the sound source frequency is 500 Hz, the raw voltage signal from the FCTH closely resembles the ideal voltage signal retaining only the 500 Hz frequency. However, at 50 Hz and 12,000 Hz sound source frequencies, the voltage waveform exhibits significant harmonic distortion. This is because these two frequencies represent the upper and lower response limits of the FCTH, resulting in a reduced output voltage amplitude. Consequently, the noise signal causes a relatively noticeable interference to the voltage waveform. However, the FFT plot reveals that the dominant frequency of the raw waveform aligns with the sound source frequency, indicating the FCTH’s capability to detect underwater acoustic signals within this frequency range.

The receiving sensitivity of a hydrophone represents its acoustic-to-electrical conversion characteristics, primarily referring to free-field sensitivity and sound pressure sensitivity. Free-field sensitivity generally denotes the ratio of the open-circuit voltage of the hydrophone, when excited by a fixed-frequency sinusoidal signal under free-field environmental conditions, to the sound pressure at the center of the hydrophone’s position within the initial sound field. Sound pressure sensitivity denotes the ratio of the open-circuit voltage at the hydrophone’s output terminal to the actual sound pressure acting upon the hydrophone under specified environmental conditions. Considering the indoor hydroacoustic test setup constructed, the sound pressure sensitivity $M_{P}$ of the FCTH is measured here, with its calculation expression given by Ref. [57]:

(6) $$M_{P}=\frac{V}{p_{P}}$$

In the equation, V is the open-circuit voltage of the hydrophone, and $p_{P}$ is the sound pressure experienced by the hydrophone in the acoustic field.

In underwater acoustics, it is customary to specify the sensitivity of a hydrophone in dB (re: 1 V/Pa), referred to as the sensitivity level. The sensitivity level is the ratio of the hydrophone’s sensitivity to a reference sensitivity. Therefore, the expression for the sound pressure sensitivity level $S_{P}$ is:

(7) $$S_{P}=20\mathrm{lg}\left(\frac{M_{P}}{M_{r}}\right)$$

In the equation, $M_{r}$ represents the reference sensitivity, with a magnitude of 1 V/Pa.

Hydrophone sensitivity calibration generally employs two methods: primary calibration (absolute calibration) and secondary calibration (relative calibration). Primary calibration utilizes calibrated amplifiers and oscillators, offering high precision but involving cumbersome procedures and complex testing. The secondary calibration method employs a calibrated standard hydrophone as the reference unit, simplifying the calibration process. This paper adopts the displacement comparison method within the secondary calibration approach. The calibration principle of displacement comparison is as follows: by sequentially placing the standard hydrophone and the test hydrophone at the same position within the acoustic field, the ratio of their sensitivities equals the ratio of their open-circuit voltages. During the experiment, a standard commercial hydrophone with known sensitivity serves as the reference. It and the test hydrophone are alternately placed at the same position in the underwater acoustic field. Under the same sound source excitation, the ratio of their open-circuit voltages is measured, enabling the calculation of the sensitivity level of the test hydrophone. The sensitivity formula [58] for the test hydrophone is:

(8) $$S_{p-test}=20\mathrm{lg}\frac{V_{test}}{V_{ref}}+S_{p-ref}$$

In the equation, $S_{p-test}$ is the sensitivity of the test hydrophone, $S_{p-ref}$ is the sensitivity of the standard commercial hydrophone, $V_{test}$ is the open-circuit voltage of the test hydrophone, and $V_{ref}$ is the open-circuit voltage of the standard commercial hydrophone.

In previous experimental tests, the output voltage values of the RHS-30 standard hydrophone and the FCTH at one-third octave band sampling points were already known. Therefore, the output voltage of the RHS-30 standard hydrophone was measured separately under 50 Hz and 12,000 Hz acoustic waves. According to the factory test report for the RHS-30 hydrophone, the sensitivity values within the 50–12,000 Hz. For computational convenience, it is uniformly considered as −192 dB. The sensitivity of the FCTH was calculated using the sensitivity formula, yielding the data shown in Table 2. This includes the output voltage values and sensitivity values of the FCTH at the upper and lower frequency limits and at the one-third octave band sampling points.

Testing yielded output voltage values for both the standard hydrophone and the FCTH across the 50 Hz to 12,000 Hz frequency range. Table 2 presents the output voltage values and sensitivity values for the FCTH. To provide a more intuitive comparison of the performance between the two devices, frequency response tests were conducted on the standard hydrophone and the FCTH under constant sound pressure of 1 Pa. The test results are shown in Figure 3D(I). When the acoustic frequency is between 315 Hz and 500 Hz, the output voltage of the FCTH is greater than that of the standard hydrophone. The standard hydrophone exhibits a sound pressure sensitivity of −192 dB across the 50 Hz to 12,000 Hz range. Consequently, the sensitivity of the FCTH can be calculated using the formula, with its sensitivity curve depicted in Figure 3D(II). The sensitivity of the FCTH exhibits significant fluctuations within the 50 Hz to 12,000 Hz frequency range, with a maximum value of −174.6 dB and a minimum value of −221.4 dB. However, within the 315 Hz to 500 Hz frequency range, the sensitivity of the FCTH surpasses that of the standard hydrophone. Additionally, to ensure the accuracy of test data and the reliability of sensitivity calculations, this study conducted background noise tests, thereby effectively eliminating the impact of environmental noise and systematic errors on measurement results, as shown in the Figure S2.

To evaluate measurement repeatability and device-to-device variation, three independent FCTH devices (FCTH-A, FCTH-B, and FCTH-C) were fabricated and each measured three times under identical acoustic excitation conditions. The mean output voltage, standard deviation (SD), and 95% confidence interval (CI) were calculated across all nine measurements for representative frequencies from 50 Hz to 12,000 Hz. As shown in Table S1, the results show that the coefficient of variation (CV) remains below 6% across the entire frequency range, indicating good repeatability and stable sensitivity among devices.

To measure the directivity of a FCTH, a 500 Hz sine wave with 1 Pa sound pressure was selected as the excitation signal. The starting point was defined as the gap between PTFE films, with the initial angle set at 0°. The sound source rotated a full 360°, with the output voltage signal of each sensor measured every 20° of rotation. Directivity is another crucial parameter for hydrophones, representing their sensitivity to sound sources from different directions. It is typically described using a directivity diagram in polar coordinates, which illustrates how the hydrophone’s output signal varies with the direction of the sound source. In directivity measurement experiments, data normalization is required, with the formula [59] given as follows:

(9) $$L=20\mathrm{lg}\left(\frac{V_{\theta }}{V_{\max}}\right)$$

In the equation, θ represents the rotation angle of the sound source, $V_{\theta }$ denotes the output voltage of the tested hydrophone at any rotation angle, and $V_{\max}$ represents the maximum output voltage of the tested hydrophone.

Ultimately, the directionality of the FCTH is shown in Figure 3E. It can be observed that the FCTH exhibits horizontal omnidirectionality, demonstrating nearly equal sensitivity to sound sources originating from all horizontal directions. The FCTH can detect acoustic signals from all directions, further enhancing the underwater acoustic detection performance of triboelectric hydrophones.

To assess stability, the FCTH was continuously submerged underwater, with daily frequency response range tests conducted, as shown in Figure 3F. Within the first 48 h, the frequency response curve exhibited minimal variation. However, over time, the output voltage at different frequency points progressively decreased. Analysis revealed that although dimethyl silicone oil is unlikely to react with vulcanized latex balloons, prolonged immersion caused swelling of the latex material. This swelling gradually reduced the balloon’s elasticity, leading to a progressive decline in the FCTH’s performance. Silicone oil has a limited swelling effect on polar rubber materials such as nitrile or neoprene. This property can help improve material stability. Future iterations could use custom polar rubber sleeves to further enhance performance.

### 3.3. Acoustic Detection Testing of the FCTH

#### 3.3.1. Detect Airborne Sound Pressure Signals

**Figure 4:**
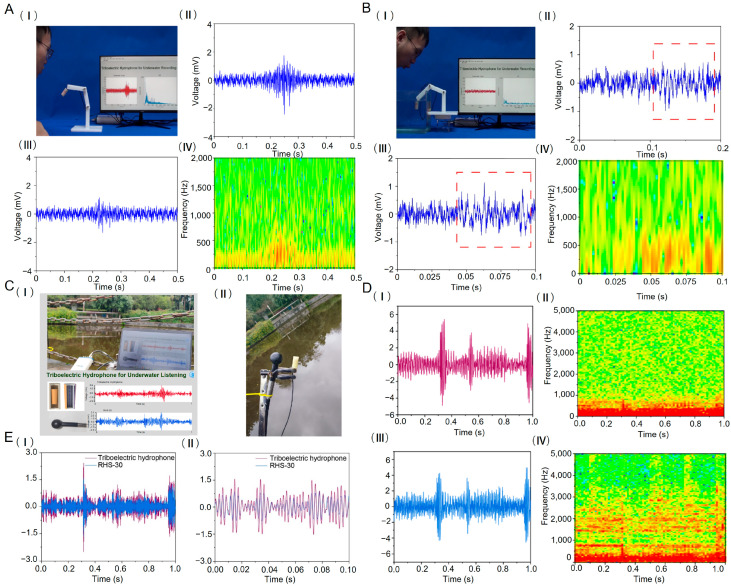
(**A**) Air blowing test: (**I**) Experimental test environment diagram: (**II**) Output voltage of the frustum-cone triboelectric hydrophone; (**III**) Output voltage of the frustum-cone triboelectric hydrophone; (IV) STFT images of voltage signal. (**B**) Water surface blowing test: (**I**) Experimental test environment diagram; (**II**) Output voltage of the frustum-cone triboelectric hydrophone; (**III**) Output voltage of the frustum-cone triboelectric hydrophone; (IV) STFT images of the voltage signal. (**C**) Outdoor underwater acoustic detection: (**I**) Outdoor hydroacoustic test site; (**II**) Installation diagram of the hydrophones. (**D**) Output voltage characteristic of the hydrophones: (**I**) Output voltage of the frustum-cone triboelectric hydrophone; (**II**) STFT image of the frustum-cone triboelectric hydrophone; (**III**) Output voltage of the standard hydrophone; (IV) STFT image of the standard hydrophone. (**E**) Comparison of waveform: (**I**) bandpass filtered voltage at 315–500 Hz for the frustum-cone triboelectric hydrophone and the standard hydrophone; (**II**) bandpass filtered voltage at 315–500 Hz for the frustum-cone triboelectric hydrophone and the standard hydrophone in 0–0.1 s.

The detection capability of the FCTH for weak acoustic pressure signals was validated by capturing airflow signals in non-acoustically impedance-matched conditions. Figure 4A(I) illustrates the experimental setup, where the FCTH was secured to a 3D-printed free-moving frame using epoxy adhesive to prevent sensor displacement under airflow and ensure experimental accuracy. During testing, researchers blew air toward the sensor from approximately 10 cm away. The output voltage signal from the triboelectric hydrophone was transmitted to the LabVIEW host computer via an NI USB-4431 acquisition card. The host interface then displayed real-time output voltage waveforms and voltage spectrum plots. Figure 4A(II,III) show two output voltage signals generated by the FCTH after airflow impact. Noticeable fluctuations in the voltage waveform indicate that the hydrophone captured the airflow signal. As shown in Figure 4A(IV), applying short-time Fourier transform (STFT) processing to the voltage waveform in 4A(III) reveals that the signal frequencies generated after the airflow signal reaches the surface of the FCTH’s guide vane are primarily concentrated below 500 Hz. This confirms that the FCTH can detect low-frequency, weak sound pressure signals.

#### 3.3.2. Detect Acoustic Pressure Signals in Water

After demonstrating the ability of the FCTH to detect faint airborne acoustic signals, a comparative test was designed to evaluate its detection of faint underwater acoustic signals. As shown in Figure 4B(I), the FCTH was fixed underwater. The researcher blew air onto the water surface surrounding the hydrophone while simultaneously displaying its output voltage signal and voltage spectrum in real-time on the host computer interface. The final experimental results, as shown in Figure 4B(II,III), reveal subtle changes in the output voltage signal of the FCTH within the red dashed box after the airflow impacted the water surface. This indicates that the FCTH successfully captured the acoustic pressure signal in the water. As shown in Figure 4B(IV), STFT processing of the voltage signal in 4B(III) revealed that the signal frequencies generated by the airflow impact on the water surface were primarily concentrated below 750 Hz. Comparing the two sets of experiments, when air was blown directly onto the FCTH, the sensor’s output voltage signal was significantly more pronounced in both voltage amplitude and waveform characteristics than when air was blown onto the water surface surrounding the hydrophone. This occurs because the acoustic pressure variations generated by the airflow directly impact the FCTH. In contrast, blowing air toward the water surface diverts energy into work done on the water, reducing the signal energy received by the hydrophone. Nevertheless, these results demonstrate the FCTH’s capability to detect low-frequency, faint underwater acoustic signals.

#### 3.3.3. Underwater Testing in Outdoor Water Bodies

To validate the underwater acoustic detection capabilities of the FCTH in real aquatic environments, field tests were conducted in outdoor water bodies. Both the RHS-30 standard hydrophone and the FCTH were simultaneously deployed to detect underwater music. The test site is shown in Figure 4C(I). Experimental conditions were identical to those of the previous outdoor acoustic detection tests for both FCTH and are not repeated here. The figure also displays the host computer program interface, which can simultaneously show the output voltage signals from both the standard hydrophone and the FCTH. Figure 4C(II) shows the RHS-30 standard hydrophone and the FCTH secured with tension clamps. By adjusting the sensor positions, both hydrophones were positioned at the same height.

Under the excitation of underwater music, the output voltage curve of the FCTH is shown in Figure 4D(I), while that of the standard hydrophone is shown in Figure 4D(III). The output voltage waveform profiles of both devices are generally consistent with similar voltage amplitudes, though the standard hydrophone’s waveform appears denser. This difference primarily stems from the varying sensitivities between the FCTH and the standard hydrophone. As shown in Figure 4D(II,IV), applying the STFT transformation to the raw voltage waveforms of the FCTH and standard hydrophone yielded their respective spectral relationship diagrams. The FCTH primarily captures underwater acoustic signals below 700 Hz, while the standard hydrophone can capture signals up to 3500 Hz.

To further compare acoustic detection performance, knowing that the FCTH exhibits higher sensitivity than the standard hydrophone in the 315–500 Hz frequency range, the output voltage signals were subjected to bandpass filtering in this frequency band. After filtering, the filtered voltage waveforms of the FCTH and the standard hydrophone are shown in Figure 4E(I). Due to differences in sensitivity, amplitude variations persist in the filtered voltage waveforms. To examine waveform details, Figure 4E(II) displays the filtered voltages over 0–0.1 s. The voltage waveforms exhibit highly similar trends, with the FCTH producing a larger output voltage amplitude. This indicates that the sensitivity of the FCTH exceeds that of the standard hydrophone after filtering, demonstrating its capability to detect low-frequency underwater acoustic signals in real aquatic environments.

## 4. Conclusions

This study investigates the low-frequency FCTH, comprising three components: a cylindrical polyurethane flow guide, silicone oil, and the conical triboelectric hydroacoustic transducer unit. The conical triboelectric hydroacoustic transducer unit uses a diaphragm and a perforated tube to form a resonance structure. This design further improves the hydroacoustic directivity and expands the frequency response range of the triboelectric hydrophone. Simultaneously, the FCTH incorporates a miniaturized design that significantly reduces the sensor’s dimensions. A theoretical analysis model was established using acoustic principles, thin-film vibration theory, and the triboelectric effect. Simulation software was employed to model the thin-film vibration modes and sensor resonant frequencies. Testing confirmed that the FCTH operates within a frequency range of 50 Hz to 12,000 Hz, exhibits horizontal omnidirectionality, and demonstrates sensitivity exceeding that of standard hydrophones in the frequency band of 315–500 Hz. Acoustic signal acquisition tests conducted in air, indoor water, and outdoor water environments validated the FCTH’s potential for underwater acoustic detection.

## Figures and Tables

**Table 1:** Output voltages of frustum-cone triboelectric hydrophones at 1/3 octave point (mV).

F (Hz)	Structures I	Structures II	F (Hz)	Structures I	Structures II
50	2.2	1.6	1000	30.1	3.6
63	2.4	2.1	1250	31.2	3
80	3.5	3.3	1600	34.3	2.5
100	15.8	24.5	2000	42.7	2.3
125	24.7	28.2	2500	10.4	2.1
160	25.6	27.6	3150	6.1	N/A
200	47.3	30.9	4000	4.6	N/A
250	27.6	28.1	5000	3.5	N/A
315	97.1	23.2	6300	5.2	N/A
400	274	22.9	8000	4.3	N/A
500	129.4	19.3	10,000	8	N/A
630	45.4	13.9	12,000	1.7	N/A
800	5.8	4.5	N/A	N/A	N/A

**Table 2:** Output voltages and sensitivity values of round table triboelectric hydrophone.

F (Hz)	V (mV)	S (dB)	F (Hz)	V (mV)	S (dB)
50	2.2	−209.9	1000	30.1	−197.3
63	2.4	−216	1250	31.2	−197.7
80	3.5	−221.4	1600	34.3	−202.4
100	15.8	−212.6	2000	42.7	−196
125	24.7	−209.5	2500	10.4	−211.5
160	25.6	−205.5	3150	6.1	−213.6
200	47.3	−194.5	4000	4.6	−203.3
250	27.6	−195.7	5000	3.5	−201.5
315	97.1	−186.1	6300	5.2	−197
400	274	−174.6	8000	4.3	−196.7
500	129.4	−183.7	10,000	8	−195.6
630	45.4	−192.7	12,000	1.7	−206
800	5.8	−213.3	N/A	N/A	N/A

## Data Availability

The original contributions presented in this study are included in the article/Supplementary Materials. Further inquiries can be directed to the corresponding authors.

## Supplementary Materials

The following supporting information can be downloaded at: https://www.mdpi.com/article/10.3390/nano15231765/s1, Figure S1: Electron microscope scan of the PTFE film (scale bar: 500 nm); Table S1: Repeatability results of FCTH under identical acoustic excitation (n = 9); Figure S2: The noise floor; Table S2: Key Materials, Dimensions, and Tolerances of the Triboelectric Hydrophone.
