# Supplementary material for: Miniaturized Frustum-Cone Triboelectric Hydrophone Based on a Thin Film Perforated Tube Structure
Source: Nanomaterials (Basel). 2025 Nov 25;15(23):1765. doi: 10.3390/nano15231765 (PMC12693220; doi:10.3390/nano15231765)
Supplement: Supplementary file 1 [file nanomaterials-15-01765-s001.zip › nanomaterials-3913471-supplementary.pdf]

To further increase the effective contact area of the friction layer and enhance the output electrical signal, nanowire arrays were fabricated on the surface of PTFE film. The fabrication process is as follows: First, the PTFE membrane was cleaned with menthol, isopropanol, and deionized water. The dried PTFE membrane was then placed into the reaction chamber of the reactive ion etcher. After closing the chamber door, experimental parameters were set: oxygen gas flow at 30 sccm, RF power at 100 W, and a process duration of 5 minutes. Vacuum pumping commenced, and etching began when the ionization unit reached 6-2 Pa, yielding the etched PTFE membrane.

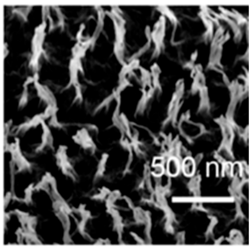

Figure S1. Electron microscope scan of the PTFE film (scale bar: 500 nm)

To evaluate measurement repeatability and device-to-device variation, three independent FCTH devices (FCTH-A, FCTH-B, and FCTH-C) were fabricated and each measured three times under identical acoustic excitation conditions. The mean output voltage, standard deviation (SD), and 95% confidence interval (CI) were calculated across all nine measurements for representative frequencies from 50 Hz to 12,000 Hz. As shown in Table S1, the results show that the coefficient of variation (CV) remains below 6% across the entire frequency range, indicating good repeatability and stable sensitivity among devices.

Table S1. Repeatability results of FCTH under identical acoustic excitation (n = 9).

F (Hz)	Mean V (mV)	SD	95% CI (mV)	Mean S (dB)	SD	95% CI (dB)
50	2.21	0.11	±0.07	-209.8	1.2	±0.8
100	15.7	0.8	±0.5	-212.5	1.1	±0.7
200	47.6	2.0	±1.3	-194.6	0.9	±0.6
315	97.0	4.9	±3.2	-186.0	1.0	±0.6
400	274.5	11.0	±7.2	-174.7	0.8	±0.5
500	130.0	6.3	±4.1	-183.8	0.9	±0.6
1000	30.3	1.6	±1.0	-197.2	0.8	±0.5
2000	42.5	2.3	±1.5	-195.9	0.7	±0.5
4000	4.7	0.2	±0.1	-203.4	1.1	±0.7
6300	5.1	0.3	±0.2	-197.1	0.9	±0.6
12000	1.8	0.1	±0.07	-206.1	0.8	±0.5

To ensure the accuracy of test data and the reliability of sensitivity calculations, this study conducted background noise tests, thereby effectively eliminating the impact of environmental noise and systematic errors on measurement results, as shown in the figure S2.

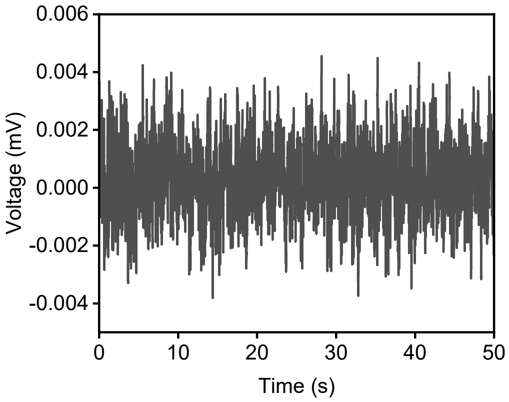

Figure S2. The noise floor.

Table S2. Key Materials, Dimensions, and Tolerances of the Triboelectric Hydrophone

Material	Dimension	Tolerance (mm)
Polyurethane cylindrical fairing	Diameter: 17 mm; Thickness: 2 mm; Total height: 35 mm	±0.05
Resin hollow cylindrical frame	Diameter: 10 mm; Lower-end outer diameter: 8 mm; Total height: 20 mm	±0.05
ITO-coated PET film	Thickness: 50 μm (Surface resistance: 150 Ω)	±0.01
PTFE film	Thickness: 50 μm	±0.01
Balloon	Thickness: 0.1 mm	±0.01
